# Superiority of lymph node ratio-based staging system for prognostic prediction in 2575 patients with gastric cancer: validation analysis in a large single center

**DOI:** 10.18632/oncotarget.9714

**Published:** 2016-05-30

**Authors:** Lin-Yong Zhao, Chang-Chun Li, Lu-Yu Jia, Xiao-Long Chen, Wei-Han Zhang, Xin-Zu Chen, Kun Yang, Kai Liu, Yi-Gao Wang, Lian Xue, Bo Zhang, Zhi-Xin Chen, Jia-Ping Chen, Zong-Guang Zhou, Jian-Kun Hu

**Affiliations:** ^1^ Department of Gastrointestinal Surgery, West China Hospital, Sichuan University, Chengdu, Sichuan, China; ^2^ Laboratory of Gastric Cancer, State Key Laboratory of Biotherapy/Collaborative Innovation Center of Biotherapy, West China Hospital, Sichuan University, Chengdu, Sichuan, China; ^3^ West China School of Pharmacy, Sichuan University, Chengdu, Sichuan, China

**Keywords:** gastric cancer, lymph node ratio, staging, prediction

## Abstract

This study aimed to evaluate the prognostic significance of node ratio (Nr), the ratio of metastatic to retrieved lymph nodes, and to investigate whether a modified staging system based on Nr can improve prognostic ability for gastric cancer patients following gastrectomy. A total of 2572 patients were randomly divided into training set and validation set, and the cutoff points for Nr were produced using X-tile. The relationships between Nr and other clinicopathologic factors were analyzed, while survival prognostic discriminatory ability and accuracy were compared among different staging systems by AIC and C-index in R program. Patients were categorized into four groups as follows: Nr0, Nr1: 0.00–0.15, Nr2: 0.15–0.40 and Nr3: > 0.40. Nr was significantly associated with clinicopathologic factors including macroscopic type, tumor differentiation, lymphovascular invasion, perineural invasion, tumor size, T stage, N stage and TNM stage. Besides, for all patients, Nr and TNrM staging system showed a smaller AIC and a larger C-index than that of N and TNM staging system, respectively. Moreover, in subgroup analysis for patients with retrieved lymph nodes < 15, Nr was demonstrated to have a smaller AIC and a larger C-index than N staging system. Furthermore, in validation analysis, Nr, categorized by our cutoff points, showed a larger C-index and a smaller AIC value than those produced in previous studies. Nr could be considered as a reliable prognostic factor, even in patients with insufficient (< 15) retrieved lymph nodes, and TNrM staging system may improve the prognostic discriminatory ability and accuracy for gastric cancer patients undergoing radical gastrectomy.

## INTRODUCTION

As one of the most common malignances, gastric cancer (GC)is nowadays the secondary leading cause of cancer-related mortality in China, in spite of a declining global incidence [1]. The identification of its prognostic factors becomes of great importance for the survival prediction of gastric cancer patients. Currently, tumor-node-metastasis (TNM) staging system, as the most commonly used staging system for gastric cancer, is applied both in the Japanese Gastric Cancer Association (JGCA) [2] and the American Joint Committee on Cancer (AJCC) [3], not only because of its discriminatory power on the prognostic difference but also due to its predictive accuracy. However, it requires examining at least 15 lymph nodes to make N staging adequately and accurately, which has limited its use in clinical practice. Fortunately, node ratio (Nr), defined as the ratio of the positive lymph nodes to the retrieved lymph nodes, needless of considering the number of retrieved lymph nodes, which was regarded as an alternative system to N staging system, has been identified as an important independent prognostic factor in majority of studies [4–12]. Nevertheless, these findings are not universally supported [13, 14], and Nr has not yet been integrated into the current staging system for gastric cancer up till now. Thus, the controversy for the prognostic significance of Nr still remains.

In light of these considerations mentioned above, we performed this study to evaluate the prognostic significance of node ratio (Nr), and to investigate whether a modified staging system, TNrM which is based on Nr, can improve prognostic discriminatory ability and predictive accuracy for gastric cancer patients undergoing gastrectomy.

## RESULT

### Correlation analysis between the clinicopathologic factors and node ratio

**Figure 1:**
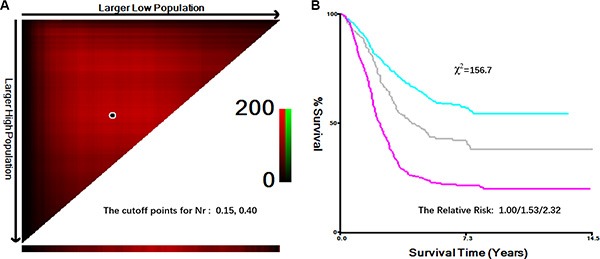
Division of patients by the cutoff points produced by X-tile plot (**A**) X-tile plots for lymph node ratio (Nr). The plots illustrate that the produced log-rank χ^2^ value stratify the node-positive patients into 3 groups by two cutoff points, 0.15 and 0.40. (**B**), survival curves generated by X-tile plots, show a strong discriminatory capacity, with a χ^2^ value of 156.7 and a relative risk ratio of 1.00/1.53/2.32.

X-tile plots, constructed in Figure 1, illustrated that the optimal cutoff points for node ratio (Nr) were 0.15 and 0.40 in node-positive patients using minimum *P* value from log-rank χ^2^ test, according to which patients were categorized into four groups, Nr0:0.0 Nr1:0.0-0.15, Nr2:0.15-0.40, Nr3: > 0.40, with the strongest discriminatory capacity.

Clinicopathologic factors were compared between the training set and validation set, and among the four groups, as shown in Table 1. There was no significant difference between the training set and validation set regarding all the clinicopathologic factors (all the p* value > 0.05), which meant that the baseline was balanced between them. Besides, both in the training and validation set, Nr stage was found to be significantly associated with macroscopic type, tumor differentiation, lymphovascular invasion, perineural invasion, tumor size, T stage, N stage and TNM stage (all the *p* value < 0.05). However, no significance was found between Nr and age, gender as well as adjuvant chemotherapy. There were significantly more patients with macroscopic type III-IV, poorly tumor differentiation, positive lymphovascular/perineural invasion, larger tumor size and advanced TNM stage in higher node ratio stages (Nr2 and Nr3) than that in lower node ratio stages (Nr0 and Nr1).

**Table 1:** Patients and clinicopathologic factors

	Training set						Validation set						P*
Factors	Nr0 (*n* = 701)	Nr1 (*n* = 441)	Nr2 (*n* = 484)	Nr3 (*n* = 477)	Total (*n* = 2103)	*P*	Nr0 (*n* = 157)	Nr1 (*n* = 115)	Nr2 (*n* = 109)	Nr3 (*n* = 91)	Total (*n* = 472)	*P*	
Gender						0.172						0.248	0.051
Male	501	308	361	333	1503		103	75	71	67	316		
Female	200	133	123	144	600		54	40	38	24	156		
Age (years)						0.067						0.131	0.059
≥ 60	308	213	209	194	924		73	57	48	52	230		
< 60	393	228	272	286	1179		84	58	61	39	242		
Tumor location						0.152						0.245	0.112
Upper third	175	104	128	117	524		39	20	25	19	103		
Middle third	155	96	96	119	466		38	39	29	22	128		
Lower third	357	232	253	230	1072		77	55	53	46	231		
≥ 2/3 stomach	14	9	7	11	41		3	1	2	4	10		
Macroscopic type						< 0.001						< 0.001	0.069
Borrmann 0–II	395	284	280	225	1184		85	83	59	17	244		
Borrmann III– IV	306	157	204	252	919		72	32	50	74	228		
Tumor differentiation						< 0.001						< 0.001	0.141
Well/Moderately	138	124	96	56	414		30	25	16	8	79		
Poorly	563	317	388	421	1689		127	90	93	83	393		
Lymphovascular invasion						0.008						0.003	0.083
Negative	437	292	305	275	1311		90	82	60	42	274		
Positive	264	147	179	202	792		67	33	49	49	198		
Perineural invasion						0.004						< 0.001	0.054
Negative	560	374	377	368	1679		121	91	69	67	358		
Positive	141	67	107	109	424		36	14	40	24	114		
Adjuvant chemotherapy						0.087						0.271	0.090
Present	254	181	195	207	837		47	45	44	32	168		
Absent/Unclear	447	260	299	270	1266		110	70	65	59	304		
Tumor size						< 0.001						< 0.001	0.102
≤ 4.5 cm	234	191	178	99	702		51	54	49	17	171		
4.5-7.5 cm	330	188	230	241	989		71	49	43	33	196		
≥ 7.5 cm	137	62	73	140	412		35	12	17	41	105		
Retrieved lymph nodes						0.901						0.900	0.121
≥ 15	499	323	348	342	1512		119	87	84	66	356		
< 15	202	118	136	135	591		38	28	25	25	116		
T Stage						< 0.001						< 0.001	0.097
T1	42	52	27	6	127		13	17	3	6	39		
T2	81	74	60	28	243		22	29	13	2	66		
T3	57	54	41	19	171		10	7	7	6	30		
T4a	435	229	309	331	1304		91	57	79	63	290		
T4b	86	32	47	93	258		21	5	7	14	47		
N Stage						< 0.001						< 0.001	0.194
N0	701	0	0	0	701		157	0	0	0	157		
N1	0	316	68	21	405		0	75	16	15	106		
N2	0	119	216	84	419		0	26	46	3	75		
N3a	0	6	184	215	405		0	11	43	35	89		
N3b	0	0	16	157	173		0	3	4	38	45		
TNM Stage						< 0.001						< 0.001	0.066
IA	290	0	0	0	290		51	0	0	0	51		
IB	129	50	11	2	192		35	10	7	2	54		
IIA	101	64	22	3	190		25	10	3	2	40		
IIB	92	47	36	12	187		25	9	8	4	46		
IIIA	82	171	79	37	369		20	66	6	11	103		
IIIB	7	92	167	75	341		1	17	48	12	78		
IIIC	0	17	169	348	534		0	3	37	60	100		

### Identification of risk factors and multicollinearit analysis for node ratio

As illustrated in Table 2, logistic regression analysis was performed to determine the risk factors for Nr. In the univariate analysis, the involved factors significantly consisted of clinicopathologic factors, such as age (OR = 1.165, *p* = 0.022), tumor location (OR = 0.382, *p* = 0.003), macroscopic type (OR = 1.430, *p* < 0.001), tumor differentiation (OR = 1.697, *p* < 0.001), lymphovascular invasion (OR = 1.436, *p* = 0.023), perineural invasion (OR = 1.037, p = 0.052), tumor size (OR = 1.541, *p* = 0.011), T stage (OR = 1.234, *p* = 0.023) and N stage (OR = 3.812, *p* < 0.001). Multivariate logistic regression model analysis indicated that tumor differentiation (OR = 1.045, *p* = 0.010), lymphovascular invasion (OR = 1.011, *p* = 0.045) and N stage (OR = 2.631, *p* < 0.001) were independent risk factors for Nr.

**Table 2:** Logistic regression analysis of the risk factors for node ratio (Nr)

Factors	Univariate analysis		Multivariate analysis	
	OR (95% CI)	*P* value	OR (95% CI)	*P* value
Gender	1.004 (0.758–1.332)	0.187	-	-
Age	1.165 (1.022–1.328)	0.022	0.856 (0.640–1.146)	0.244
Tumor location	0.382 (0.202–0.723)	0.003	1.020 (0.861–1.208)	0.973
Macroscopic type	1.430 (1.252–1.633)	< 0.001	0.807 (0.602–1.082)	0.277
Tumor differentiation	1.697 (1.433–2.010)	< 0.001	1.045 (1.008–1.321)	0.010
Lymphovascular invasion	1.436 (1.115–1.798)	0.023	1.011 (1.002–1.230)	0.045
Perineural invasion	1.037 (0.931–1.041)	0.052	-	-
Adjuvant chemotherapy	0.843 (0.665–1.210)	0.310	-	-
Tumor size	1.541 (1.326–1.791)	0.011	0.903 (0.667–1.221)	0.507
Retrieved lymph node	0.923 (0.771–1.132)	0.123	-	-
T Stage	1.234 (1.012–1.991)	0.023	1.085 (0.910–1.236)	0.064
N Stage	3.812 (2.467–4.943)	< 0.001	2.631 (1.912–3.676)	< 0.001

OR: Odds Ratio; CI: Confidence Interval.

**Figure 2:**
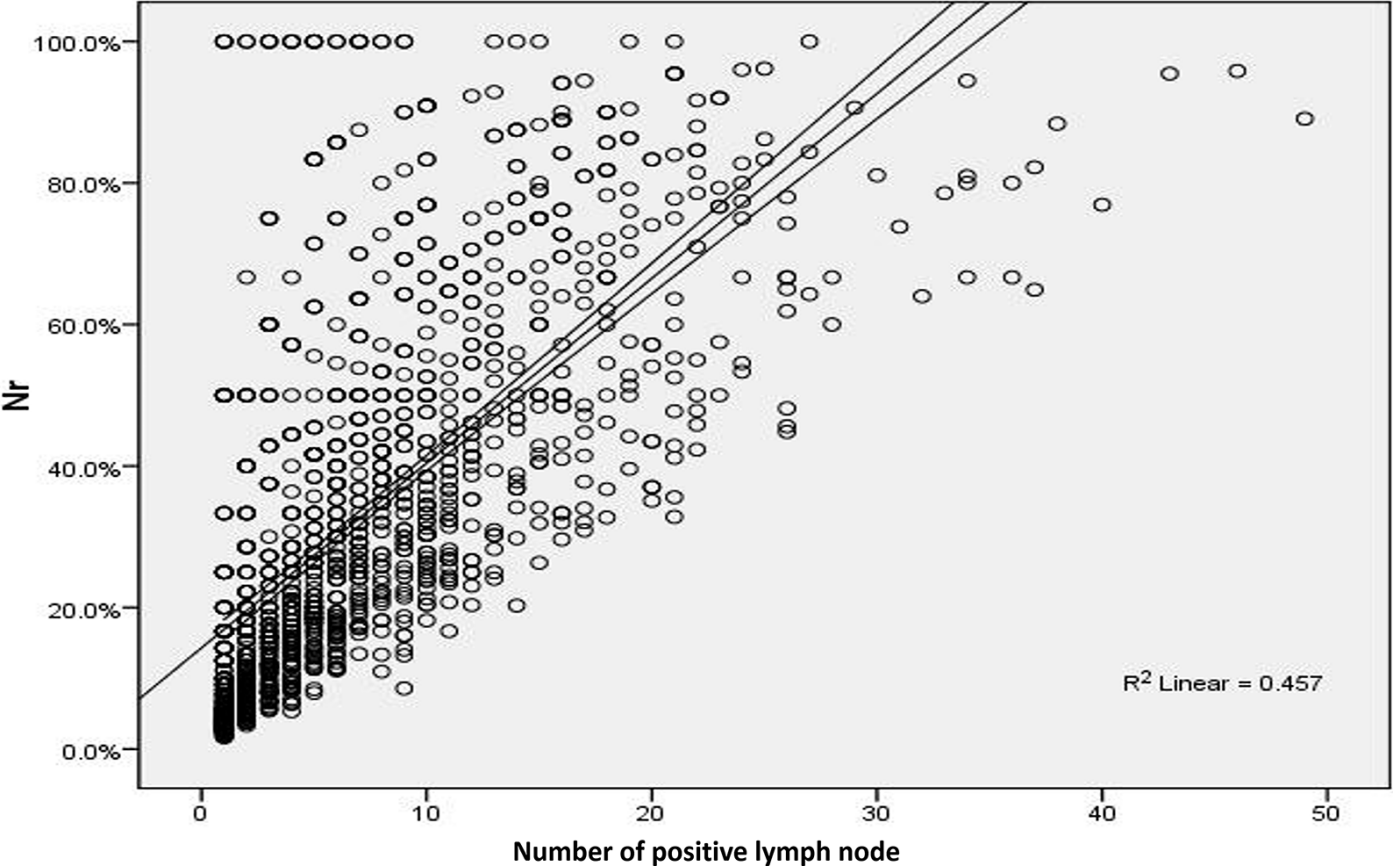
Positive linear correlation between the number of positive lymph node and Nr (R^2^ = 0.457) in scatter spots

In order to assess the multicollinearity between Nr and these independent factors identified above, spearman correlation analyses were performed in Table 3, demonstrating that N stage was correlated with Nr (*r* = 0.724, *p* < 0.001), while tumor differentiation, lymphovascular invasion and T stage showed no correlation with Nr (all the *r* value < 0.3). Additionally, scatter spots in Figure 2, suggested that there was positive linear correlation between the number of positive lymph node and Nr (*R* ^2^ = 0.457).

**Table 3:** Spearman correlation analysis of the multicollinearity for node ratio (Nr)

Factors	Correlation coefficient (r)	*P* value
Tumor differentiation	0.166*	< 0.001
Lymphovascular invasion	0.214*	< 0.001
T Stage	0.290*	< 0.001
N Stage	0.724*	< 0.001

* Correlation is significant at the 0.01 level (2 tailed);

r: correlation coefficient; |r| < 0.3: no correlation; 0.3 ≥ |r|: correlation exists.

### Univariate and multivariate analyses of factors associated with patients' prognosis

As shown in Table 4, the 5-year overall survival rates (5-y OS) for these four Nr stages were 72.5%, 63.4%, 46.9% and 22.6%, respectively, whereas the 5-y OS for different TNrM stages were 82.5%, 80.8%, 73.4%, 61.8%, 57.0%, 41.3% and 22.4%, respectively. Univariate analysis with cox regression model in Table 5 showed clinicopathologic factors, including gender, age, macroscopic type, tumor differentiation, lymphovascular invasion, perineural invasion, adjuvant chemotherapy, tumor size, T stage and N stage as well as Nr stage, were significant prognostic factors for patients. However, multivariate analysis indicated that only age (HR = 0.751, *p* < 0.001), tumor size (HR = 1.181, *p* = 0.006), T stage (HR = 1.271, *p* < 0.001), N stage (HR = 1.209, *p* = 0.003) and Nr stage (HR = 1.413, *p* < 0.001) were independent prognostic factors for gastric cancer patients, after adjustment of the confounding factors.

**Table 4:** Prognostic prediction by Kapplan–Meier survival analyses

Factors	3–year OS (%)	5–year OS (%)	MS (months)	AIC	C–index	Log rank χ^2^ value	*P*
N stage				898.7	0.687	151.1	< 0.001
N0	85.2	72.5	124.1 (7.5–176.0)				
N1	76.1	63.4	113.0 (3.5–174.0)				
N2	67.1	52.8	99.5 (1.4–172.4)				
N3a	42.3	27.5	29.9 (0.9–160.0)				
N3b	26.5	20.4	23.0 (0.3–172.7)				
Nr stage				880.1	0.696	156.7	< 0.001
Nr0	85.2	72.5	124.1 (7.5–176.0)				
Nr1	78.3	63.4	106.0 (1.4–160.0)				
Nr2	58.4	46.9	54.9 (0.3–174.0)				
Nr3	30.4	22.6	26.1 (0.9–172.7)				
TNM stage				872.3	0.754	161.7	< 0.001
IA	92.6	82.5	134.2 (9.3–156.2)				
IB	87.3	77.2	121.2 (1.4–172.4)				
IIA	81.1	71.6	101.3 (0.3–174.0)				
IIB	79.8	60.6	92.7 (4.0–176.0)				
IIIA	66,2	55.4	82.6 (5.4–157.2)				
IIIB	55.9	46.2	46.6 (0.9–150.3)				
IIIC	33.1	22.2	25.4 (0.8–172.7)				
TNrM stage				850.4	0.799	182.3	< 0.001
IA	92.6	82.5	134.2 (9.3–165.2)				
IB	90.4	80.8	112.5 (6.5–176.0)				
IIA	85.0	73.4	98.4 (1.4–160.9)				
IIB	79.5	61.8	84.6 (0.3–173.1)				
IIIA	64.3	57.0	65.4 (0.8–161.8)				
IIIB	56.4	41.3	47.3 (0.9–175.6)				
IIIC	30.2	22.4	24.0 (0.6–172.3)				

OS: overall survival ; MS: median survival time; AIC: Akaike Information Criterion value; C–index: concordance index.

**Table 5:** Univariate and multivariate analyses of the patients' clinicopathologic factors by Cox regression model

Factors	Univariate analysis		Multivariate analysis	
	HR (95% CI)	*P* value	HR (95% CI)	*P* value
Gender	0.825 (0.691–0.984)	0.033	0.808 (0.732–1.091)	0.301
Age	0.744 (0.637–0.869)	< 0.001	0.751 (0.611–0.881)	< 0.001
Tumor location	0.933 (0.852–1.022)	0.138	–	–
Macroscopic type	1.329 (1.137–1.553)	< 0.001	1.016 (0.872–1.221)	0.754
Tumor differentiation	1.109 (1.032–1.257)	0.041	1.012 (0.819–1.273)	0.288
Lymphovascular invasion	1.246 (1.129–1.478)	< 0.001	1.138 (0.932–1.431)	0.064
Perineural invasion	1.051 (1.002–1.171)	0.087	–	–
Adjuvant chemotherapy	1.244 (1.112–1.653)	0.032	1.104 (0.912–1.412)	0.136
Tumor size	1.682 (1.461–1.796)	< 0.001	1.181 (1.041–1.353)	0.006
Retrieved lymph node	0.893 (0.652–1.012)	0.211	–	–
T stage	1.539 (1.404–1.686)	< 0.001	1.271 (1.151–1.423)	< 0.001
N stage	1.593 (1.473–1.723)	< 0.001	1.209 (1.013–1.389)	0.003
Nr stage	1.856 (1.674–2.056)	< 0.001	1.413 (1.147–1.698)	< 0.001

HR: Hazard Ratio; CI: Confidence Interval; – :not enter the regression model.

### Comparison and validation of different staging systems

**Figure 3:**
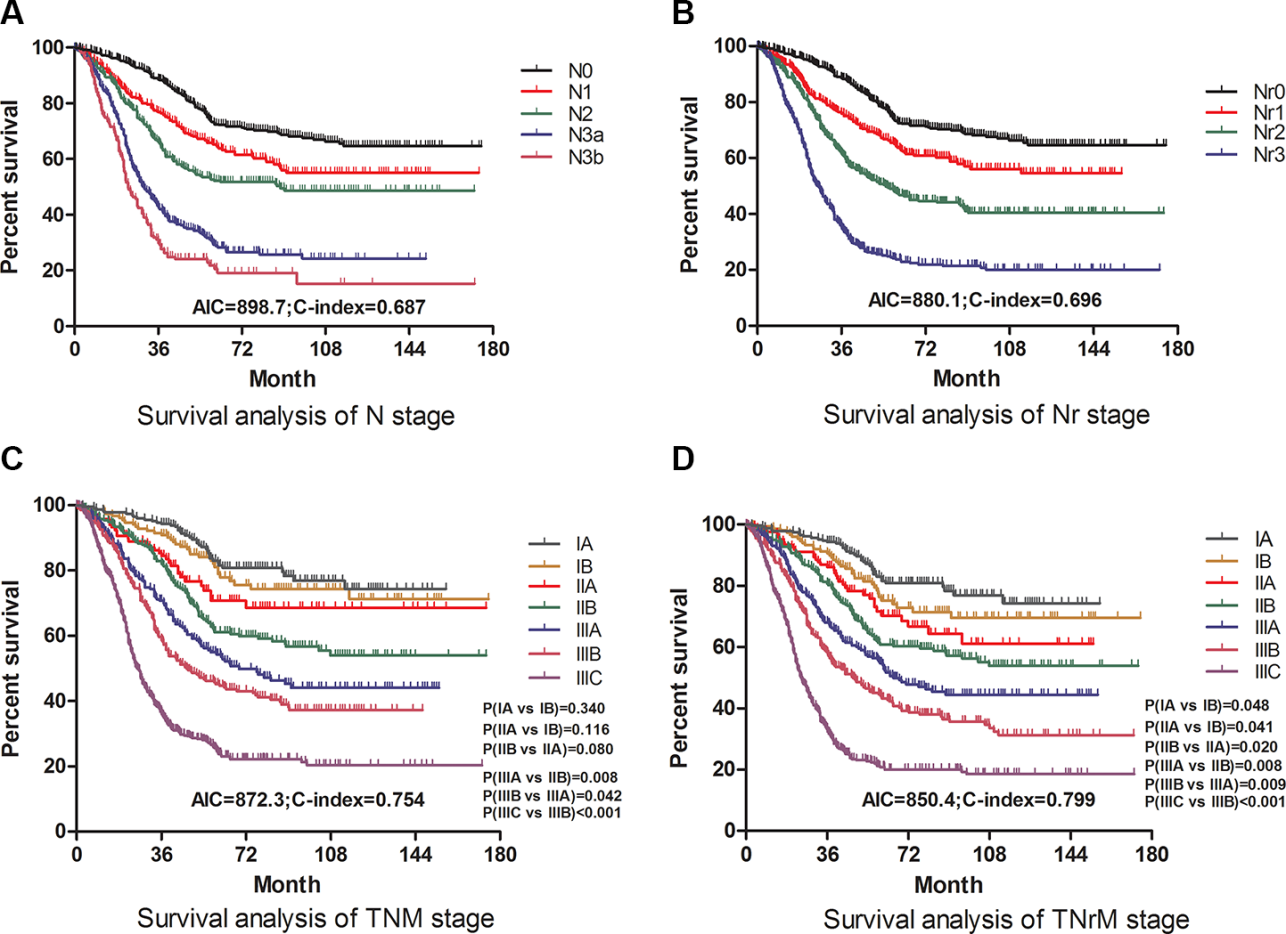
Comparative survival analysis on discriminatory ability and accuracy (**A**), survival curves of patients according to subgroups of N stage. (**B**), survival curves of patients according to subgroups of Nr stage. (**C**), survival curves of patients according to subgroups of TNM stage. (**D**), survival curves of patients according to subgroups of TNrM stage. The significance of difference between survival curves was calculated by the log-rank test.

**Figure 4:**
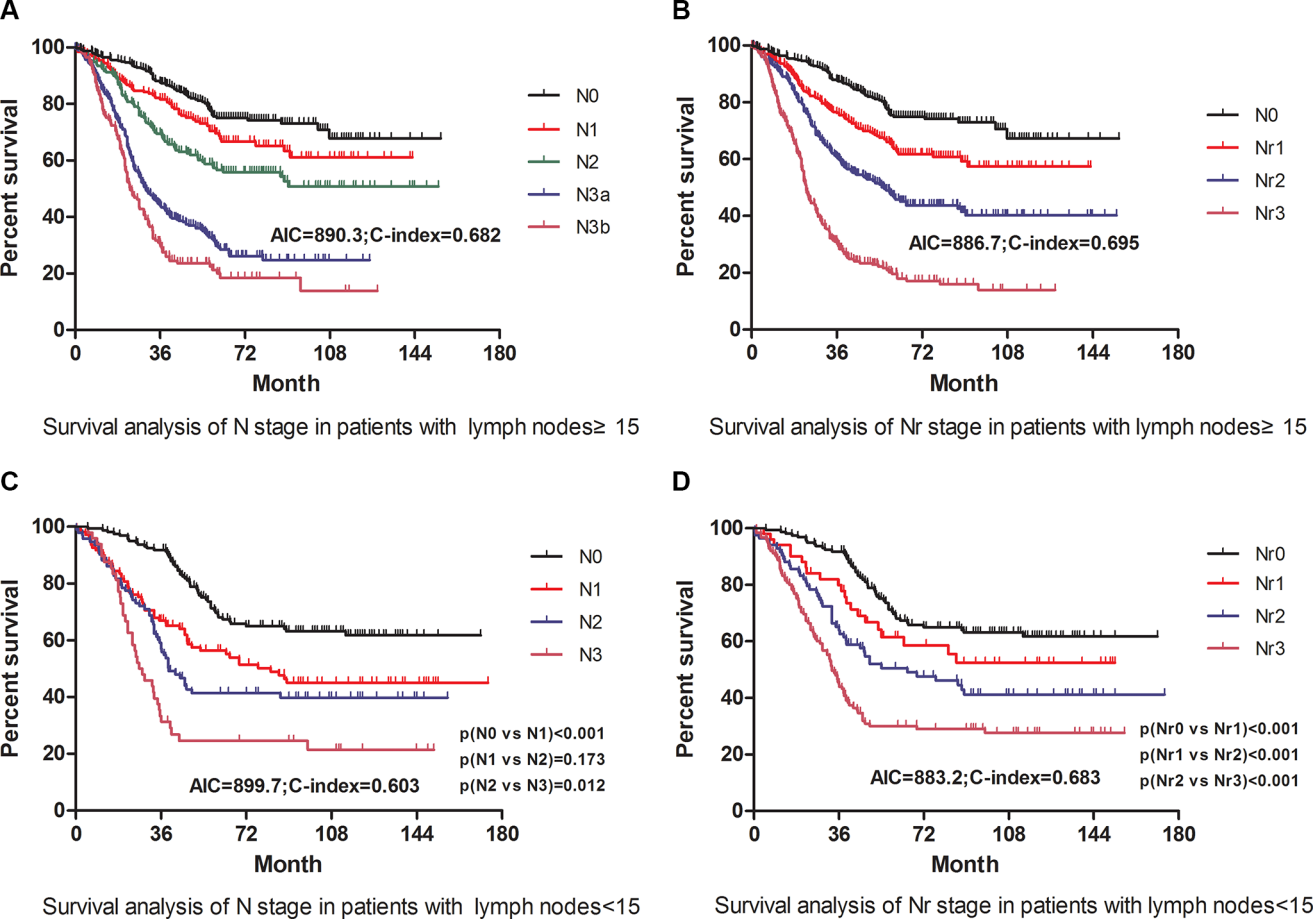
Stratified comparative survival analysis on discriminatory ability and accuracy according to the different number of retrieved lymph nodes (**A**, **B**): survival curves of patients with retrieved lymph node ≥ 15 in terms of subgroups of N and Nr stage. (**C**, **D**): survival curves of patients with retrieved lymph node < 15 in terms of subgroups of N and Nr stage. The significance of difference between survival curves was calculated by the log-rank test.

Akaike information criterion (AIC) and concordance index (C-index) values for each staging system in Table 4 were calculated to evaluate the prognostic discriminatory ability and predictive accuracy, respectively. Compared with N staging system, Nr staging system had a smaller AIC value and a larger C-index (*p* < 0.05, Figure 3A and 3B), indicating that Nr stage was advantageous to N stage in survival prediction discriminatory ability and accuracy. In addition, TNrM staging system was found to be with a larger C-index and a smaller AIC than that of current TNM staging system (*p* < 0.05), and overlapping curves were found in the TNM staging system but not in the TNrM staging system (Figure 3C and 3D), with no significant difference on survival between stage IA and IB (*p* = 0.340), stage IB and stage IIA (*p* = 0.116), stage IIA and IIB (*p* = 0.080) existing in the current TNM staging system, which illustrated that TNrM staging system had a better discriminatory ability and accuracy than that of TNM staging system in prognostic prediction. In the subgroup analysis, for patients with retrieved lymph nodes < 15, Nr staging system (AIC = 883.2; C-index = 0.683) suggested significant improvement than N staging system (AIC = 889.7; C-index = 0.603) (*p* < 0.05, Figure 4A and 4B), whereas no significant difference was found between Nr and N staging system in patients with retrieved lymph nodes ≥15 in prognostic discriminatory ability and predictive accuracy (*p* > 0.05, Figure 4C and 4D).

**Figure 5:**
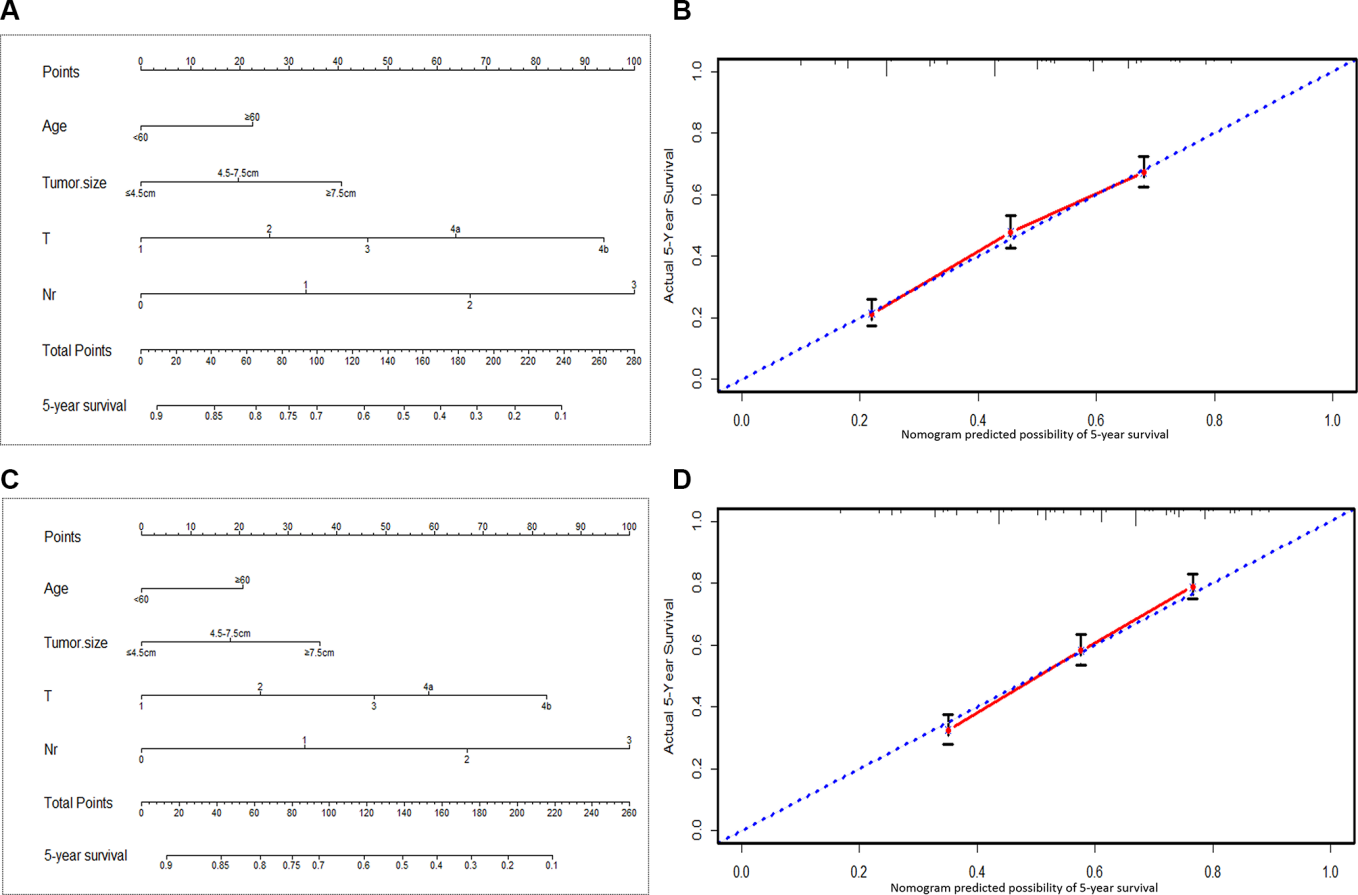
Nomogram plots and calibration curves based on Nr stage (**A**, **B**): nomogram plots and calibration curves in the training set. (**C**, **D**): nomogram plots and calibration curves in the validation set.

We also respectively applied the Nr and TNrM staging system in the validation set, found that the results were as same as that in the training set: both of Nr and TNrM staging revealed significant superiority to their counterparts, N and TNM staging system. Furthermore, nomograms were used to predict 5-year OS of patients. Both in the training set and validation set, Nr was selected as an independent prognostic factor in nomograms (Figure 5A and 5C), which was similar to those of aforementioned multivariate analysis by cox regression. Moreover, corresponding calibration curves in the two sets suggested that the predictive probability of 5-year survival were closely to the actual 5-year survival t (Figure 5B and 5D).

In order to evaluate how much improvement was gained using the cutoff points in this study, we also validated the different cutoff points reported in previous studies to create several predictive staging systems, generating various AIC values and C-indexes, as shown in Table 6. We furtherly compared them to the AIC and C-index produced in our study, and found that the cutoff points, 0, 0.15, 0.40, in our study, had a larger C-index and a smaller AIC value than those produced in previous studies (all *p* < 0.05, Table 6), illustrating the cutoff points produced in our study using X-tile were the optimal ones.

**Table 6:** Comparison and validation of different cutoff points for Nr

Authors (ref.)	Cutoff points	AIC	C-index	*P*
Melis et al [5]	0,0.30,0.60	892.1	0.701	< 0.05
Deng et al [8]	0,0.13,0.80	900.3	0.698	< 0.01
Zeng et al [16]	0,0.50,0.80	912.4	0.674	< 0.05
Wang et al [10]	0,0.067, 0.30,0.70	886.9	0.712	< 0.05
Lee et al [23]	0,0.05, 0.10,0.20, 0.30	998.8	0.675	< 0.05
Zhang et al [18]	0,0.10,0.25	1012.7	0.602	< 0.01
Wu et al [6]	0,0.20,0.50	862.5	0.772	< 0.05
Kutlu et al [9]	0,0.20,0.50	862.5	0.772	< 0.05
Zhou et al [11]	0,0.20,0.50	862.5	0.772	< 0.05
Wong et al [17]	0,0.20,0.50	862.5	0.772	< 0.05

## DISCUSSION

Although a great many studies, evaluating the prognostic significance of Nr in patients with gastric cancer, illustrated that Nr was an independent predictor and more emphases should be put on it, no agreement has been reached yet by far, due to the limitation of different cutoff points and evaluation criteria [4–11, 15–17]. Particularly, there existed no unified and well-recognized cutoff points for Nr in gastric cancer. In this present study, we applied three cutoff points: 0, 0.15, 0.40, produced by X-tile, which demonstrated better discriminatory ability and more predictive accuracy than those proposed in previous studies, and found that patients with larger Nr were companied by worse biological behavior as well as more aggressive features than patients with smaller Nr, both in the training and validation set.

Specifically, patients with larger Nr were found more frequently with the presence of advanced macroscopic type, poorly tumor differentiation, positive lymphovascular/perineural invasion, larger tumor size and deeper tumor invasion as well as wider lymph nodes metastasis. Besides, logistic regression analysis in our study showed that tumor differentiation, lymphovascular invasion and N stage were independent risk factors for Nr, suggesting that these three factors were closely associated with Nr and multicollinearity might exist between them. However, only Nr was confirmed to be positively correlated with N stage in spearman analysis, being consistent with previous studies [11, 14, 18], which was the reason why we substituted N with Nr in the current TNM staging system to come up with a modified staging system, TNrM.

We also focused on the prognostic significance of Nr. Apart from age, tumor size, T stage and N stage, Nr stage was illustrated to be independent prognostic factors for gastric cancer patients in multivariate Cox regression analysis. Comparison analysis on survival prediction illustrated that Nr staging system had a better discriminatory prognostic ability and a more predictive accuracy than that of N staging system. Although our findings were consistent with the majority of studies [6–9], Espin et al concluded that, Nr stage showed no improvement in predictive accuracy than N stage, despite that Nr and N stage were both demonstrated to be independent prognostic factors [14], which was due to the consideration that the proportion of patients with retrieved lymph nodes ≥ 15 in our study was not so much as that in Espin's study. Besides, nomogram, was also applied in our study to demonstrate the prognostic significance of independent factors on gastric cancer patients. Both in the training and validation set, the predictive accuracy of nomogram based on Nr was well demonstrated through calibration curves. But, we found that Nr but not N stage was included in the nomogram, which might due to the consideration that, Nr and N were both essential variables reflecting tumor biological features and interactive confounding effect like positive linear correlation existed between them. Moreover, taking the place of N stage in TNM staging system, Nr presented powerful survival discrimination for gastric cancer patients.

A good staging system, which is of great importance for gastric cancer patients in clinical practice, should be able to distinguish the survival difference among several subgroups of patients, and to provide accurate prognostic estimation and beneficial guidance of selecting appropriate adjuvant therapy [19]. As an powerful independent prognostic factor, the N stage in the current TNM staging system is based on the number of metastatic lymph nodes, regardless of the total number of retrieved lymph nodes in surgery. However, the prognosis of gastric cancer patients will be underestimated because of inappropriate staging in case of insufficient retrieved number of lymph nodes, especially when less than 15 lymph nodes are examined. The current TNM staging system was reported not to be an independent prognostic factor for the patients with retrieved lymph nodes fewer than 15 in a study from memorial sloan Kettering cancer Center [20]. Besides, the “stage migration” phenomenon could be observed in about 15% of patients with gastric cancer using the current TNM staging system [21]. Consequently, the prognostic value of N stage is questioned by many oncologists in light of these shortcomings mentioned above, and Nr, defined as the ratio of the metastatic lymph node to the total number of retrieved lymph nodes, regardless of lymph nodes number, is considered as an alternative option. In this present study, Nr stage was shown to have better discriminatory ability and more accurately prediction than N. Although there was no predictive difference for patients with retrieved lymph nodes ≥ 15, Nr stage revealed superiority in survival prediction for patients with retrieved lymph nodes < 15, demonstrating that Nr stage would better compensate for N stage shortcomings in gastric cancer patients, which is consistent with previous studies [10, 22, 23]. Additionally, a modified staging system, TNrM staging system based on Nr stage, predicted more accurately on overall survival by comparison of the current TNM staging system according to the findings in our study (Figure 3), which was consistent with the findings mentioned in previous investigations [6, 11, 16, 17, 24]. To show the improvement we got in this study, we also validated the various cutoff points produced in previous studies, which was not commonly done by previous authors, and found that the Nr stage, categorized by our cutoff points: 0, 0.15, 0.40 could produce the best prognostic discriminatory ability and predictive accuracy.

There were also some limitations in our study. First of all, our findings we got were just on the basis of a non-randomized retrospective single-center study, which might be observed by chance in spite of the large sample. In addition, there might be various perioperative treatment of patients which could affect the survival and interfere the evaluation of the prognostic factors, especially the preoperative therapy may lead to the downstage of the gastric cancer and that is why these patients were excluded in this study. Therefore, multicenter investigations are needed to evaluate the TNrM staging system can whether be superior to TNM staging system for the GC patients before stronger statement can be done.

In conclusion, Nr could be considered as a reliable prognostic factor, even in patients with insufficient (< 15) retrieved lymph nodes, and TNrM staging system may improve the prognostic discriminatory ability and accuracy for gastric cancer patients undergoing radical gastrectomy, which should be superior to the current TNM staging system.

## MATERIALS AND METHODS

### Patients

The West China Hospital Research Ethics Committee approved the retrospective analysis of anonymous data. Patient records were anonymized and de-identified prior to analysis, and signed patient informed consent was waived because of the retrospective nature of the analysis.

**Figure 6:**
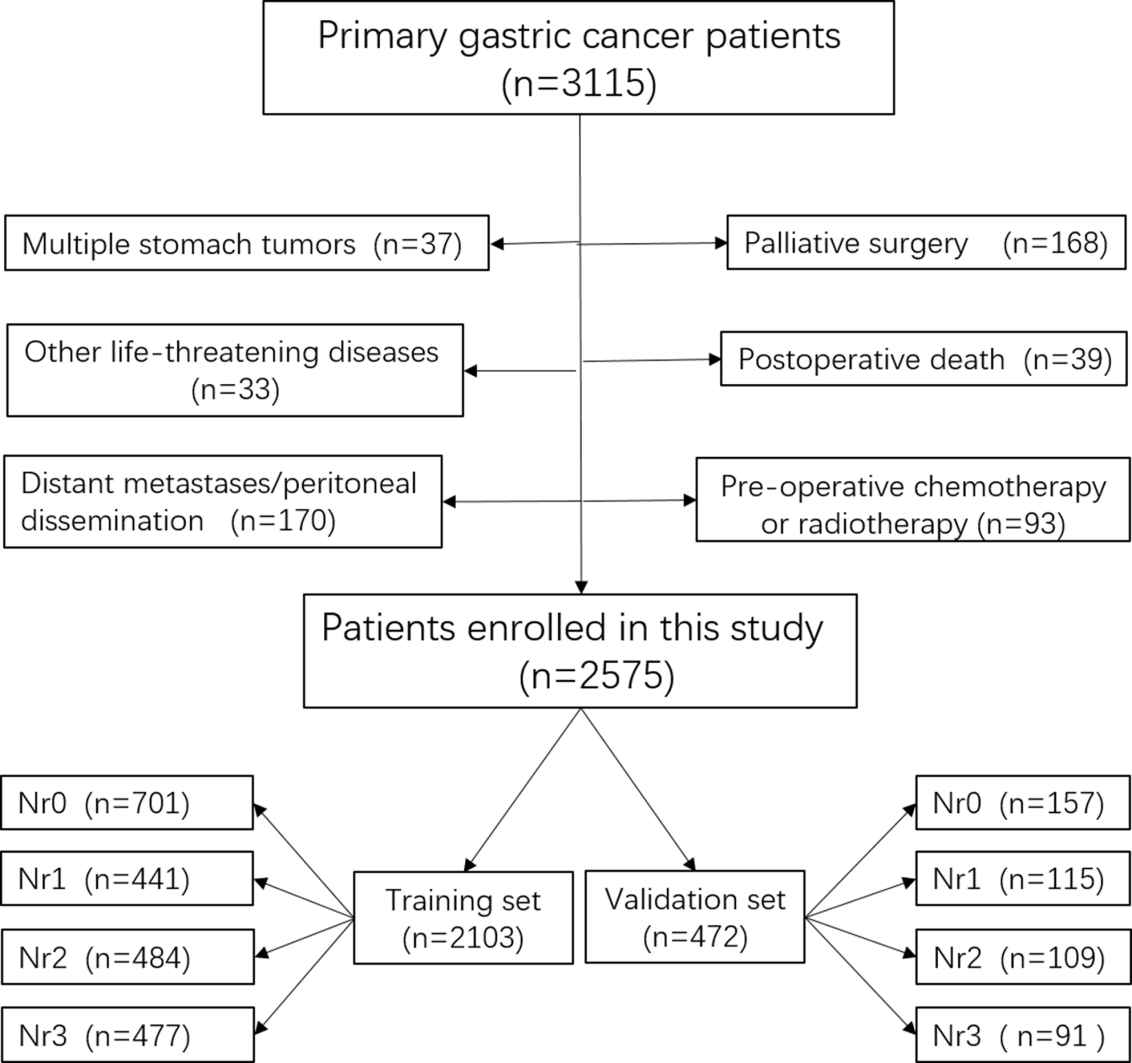
The flow chart of patients enrolled in this study

A total of 3115 consecutive GC patients who received gastrectomy in West China Hospital from January 2000 to March 2011, were retrospectively evaluated in this study. The diagnosis of primary gastric cancer for all patients was confirmed by upper gastrointestinal endoscopy and biopsy. Patients were excluded on the condition that: (1) patients who underwent palliative surgery with positive residual margins; (2) with any pre-operative chemotherapy or radiotherapy; (3) with multiple stomach tumors; (4) with another malignancy or any other life-threatening diseases diagnosed during three years prior to the operation; (5) with death due to postoperative complications in hospital; (6) with surgical findings of distant metastasis or peritoneal dissemination, or distant metastatic lymph nodes defined as M1 in JGCA [2]. Finally, 2575 patients were enrolled in this study as shown in Figure 6 and 2305 of these patients were followed up (89.50%). Patients were randomly divided into two sets using X-tile with the ratio of 4.5:1, among which 2103 patients were used as the training set, whereas 472 patients were regarded as the validation set.

The clinicopathologic characteristics including of gender, age, tumor location, macroscopic type, tumor differentiation, lymphovascular invasion, perineural invasion, postoperative adjuvant chemotherapy, tumor size, number of retrieved lymph node, T stage, N stage, TNM stage evaluated according to 7^th^ edition of AJCC TNM staging system [3] and follow-up information were collected.

### Definition of Nr and TNrM staging system

On the basis of cutoff points determined by X-tile, node ratio (Nr), the ratio between the absolute number of metastatic lymph node and the total number of lymph nodes retrieved at the time of gastric resection, was divided into four groups: Nr0 (Nr = 0.0), Nr1 (Nr:0.0–0.15), Nr2 (Nr:0.15–0.40), Nr3 (Nr ≥ 0.40), defined as Nr stage, corresponding to N0, N1, N2 and N3, respectively, in N stage. Therefore, we substituted N stage with Nr stage in TNM staging system, forming a new staging system, TNrM staging system, which was regarded as combination of T stage, Nr stage and M stage.

### Statistical analysis

X-tile program (Version 3.1.2, Yale University) was used to calculate the optimal cutoff points for Nr using minimum *P* value from log-rank χ^2^ statistics, because that it does not only play a crucial role in complicated cutoff point selection but also can randomly divide a single cohort into training set and validation set [25]. Mann-Whitney U test in the SPSS version 19.0 was applied to evaluate ranked variables, while Chi-square test was performed to analyze unordered categorical variables. Logistic regression analysis was used to analyze risk factors for Nr, whereas spearman correlation analysis was applied to evaluate the multicollinearity. Cox's proportional hazard regression model with conditional backward stepwise was displayed to univariate and multivariate survival analyses. The cumulative survival rates were calculated using the Kaplan-Meier method and life-table in the SPSS version 19.0, with subgroups compared by the log-rank test through GraphPad Prism 5. Nomogram and calibration curve were displayed using the package of Regression Modeling Strategies *(URL http://CRAN.R-project.org/package=rms)* in R (version3.1.2.*URL http://www.R-project.org/.*) Comparisons between the different staging systems for the prognostic prediction were conducted with the package of Harrell Miscellanceous *(URL http://CRAN.R-project.org/package=Hmisc.)*, in which Akaike information criterion (AIC) and concordance index (C-index) values within a cox proportional hazard regression model were calculated for each staging system to measure their discriminatory ability and accuracy, respectively. A smaller AIC valueindicated a better model for predicting outcome [8], whereas a larger C-index demonstrated a more accurate prognostic prediction [26]. The two-sided *p* value of less than 0.05 was considered to be statistically significant.
